# Reliable and Scalable SARS-CoV-2 qPCR Testing at a High Sample Throughput: Lessons Learned from the Belgian Initiative

**DOI:** 10.3390/life12020159

**Published:** 2022-01-21

**Authors:** Steven Van Vooren, James Grayson, Marc Van Ranst, Elisabeth Dequeker, Lies Laenen, Reile Janssen, Laurent Gillet, Fabrice Bureau, Wouter Coppieters, Nathalie Devos, Benjamin Hengchen, Pierre Wattiau, Sibylle Méhauden, Yvan Verlinden, Kurt Van Baelen, Theresa Pattery, Jean-Pierre Valentin, Kris Janssen, Martine Geraerts, John Smeraglia, Jan Hellemans, Pieter Wytynck, Pieter Mestdagh, Nienke Besbrugge, René Höfer, Friedel Nollet, Jo Vandesompele, Pieter De Smet, John Lebon, Emmanuel Vandewynckele, Steven Verstrepen, Wouter Uten, Arnaud Capron, Hugues Malonne, Jeroen Poels, Emmanuel André

**Affiliations:** 1 UgenTec, Inc., Cambridge, MA 02138, USA; james.grayson@ugentec.com; 2 Department of Microbiology, Immunology and Transplantation, KU Leuven, B-3000 Leuven, Belgium; marc.vanranst@uzleuven.be (M.V.R.); lies.laenen@uzleuven.be (L.L.); emmanuel.andre@uzleuven.be (E.A.); 3 Clinical Department of Laboratory medicine and National Reference Center for Respiratory Pathogens, University Hospitals Leuven, B-3000 Leuven, Belgium; els.dequeker@kuleuven.be (E.D.); reile.janssen@uzleuven.be (R.J.); 4 Biomedical Quality Assurance Research Unit, Department of Public Health and Primary Care, University of Leuven, B-3000 Leuven, Belgium; 5 Department of Microbiology, University of Liège, B-4000 Liège, Belgium; L.Gillet@uliege.be (L.G.); Fabrice.Bureau@uliege.be (F.B.); Wouter.Coppieters@uliege.be (W.C.); 6 GSK Vaccines, B-1330 Rixensart, Belgium; nathalie.i.devos@gsk.com (N.D.); benjamin.p.hengchen@gsk.com (B.H.); pierre.x.wattiau@gsk.com (P.W.); sybille.mehauden@gsk.com (S.M.); 7 Janssen Pharmaceutica N.V., Johnson & Johnson, B-2340 Beerse, Belgium; yverlind@its.jnj.com (Y.V.); kvbaele1@its.jnj.com (K.V.B.); tpattery@its.jnj.com (T.P.); 8 UCB Pharma, B-1420 Braine l’Alleud, Belgium; jean-pierre.valentin@ucb.com (J.-P.V.); kris.janssen@ucb.com (K.J.); martine.geraerts@ucb.com (M.G.); john.smeraglia@ucb.com (J.S.); 9 Biogazelle, Technologiepark Zwijnaarde, B-9052 Zwijnaarde, Belgium; Jan.hellemans@biogazelle.com (J.H.); pieter.wytynck@biogazelle.com (P.W.); pieter.mestdagh@biogazelle.com (P.M.); Nienke.besbrugge@gmail.com (N.B.); dr.rene.hofer@gmail.com (R.H.); friedel.nollet@azsintjan.be (F.N.); jo.vandesompele@biogazelle.com (J.V.); 10 CliniSys|MIPS, B-9000 Ghent, Belgium; pieter.de.smet@mips.be (P.D.S.); John.Lebon@mips.be (J.L.); Emmanuel.Vandewynckele@mips.be (E.V.); Steven.Verstrepen@mips.be (S.V.); 11 UgenTec n.v., B-3500 Hasselt, Belgium; Wouter.Uten@ugentec.com; 12 Sciensano, Belgian Institute for Health, B-1050 Brussels, Belgium; Arnaud.Capron@sciensano.be; 13 Federal Agency for Medicines and Health Products (FAGG-AFMPS), B-1210 Brussels, Belgium; hugues.malonne@fagg-afmps.be (H.M.); jeroen.poels@fagg-afmps.be (J.P.); 14 Department of Pharmacology, Pharmacotherapy and Pharmaceutical care, Faculty of Pharmacy, Université libre de Bruxelles, B-1070 Brussels, Belgium; 15 Department of Biomedical Sciences, Namur Research Institute for Life Sciences, University of Namur, B-5000 Namur, Belgium

**Keywords:** SARS-CoV-2, qPCR, lab automation, qc monitoring, high-throughput testing, data analysis

## Abstract

We present our approach to rapidly establishing a standardized, multi-site, nation-wide COVID-19 screening program in Belgium. Under auspices of a federal government Task Force responsible for upscaling the country’s testing capacity, we were able to set up a national testing initiative with readily available resources, putting in place a robust, validated, high-throughput, and decentralized qPCR molecular testing platform with embedded proficiency testing. We demonstrate how during an acute scarcity of equipment, kits, reagents, personnel, protective equipment, and sterile plastic supplies, we introduced an approach to rapidly build a reliable, validated, high-volume, high-confidence workflow based on heterogeneous instrumentation and diverse assays, assay components, and protocols. The workflow was set up with continuous quality control monitoring, tied together through a clinical-grade information management platform for automated data analysis, real-time result reporting across different participating sites, qc monitoring, and making result data available to the requesting physician and the patient. In this overview, we address challenges in optimizing high-throughput cross-laboratory workflows with minimal manual intervention through software, instrument and assay validation and standardization, and a process for harmonized result reporting and nation-level infection statistics monitoring across the disparate testing methodologies and workflows, necessitated by a rapid scale-up as a response to the pandemic.

## 1. Introduction to the Testing Initiative and Early Consortium Activities

In early 2020, the PCR-testing capacity for COVID-19 in Belgium was limited to one national reference laboratory—The National Reference Center for Respiratory Pathogens (NRC) at the University Hospitals of Leuven (UZ Leuven). Consecutively, under the supervision of the National Institute of Public Health (Sciensano) and the NRC, other clinical laboratories rapidly developed the technique, which led to the creation of a national network of clinical laboratories comprising mostly hospital and some private laboratories [1] to address the rapidly growing need for testing. University research units also joined the effort.

At the end of March 2020, the Belgian Government announced the creation of a consortium and the establishment of a national program for the upscaling of SARS-CoV-2 PCR testing with a target capacity of ≥10,000 samples per day, up from the initial daily testing capacity of 2000 samples available in Belgium in March—due to a lack of reagents and instruments [2]. To achieve throughput goals, a consolidated strategy was implemented by the Task Force under the supervision of the Federal Ministers of Health and of Digital Affairs, the Federal Agency for Medicines and Health products (FAMHP), Sciensano, and the NRC.

To increase the qPCR testing capacity nationally, universities were called upon to develop new testing methods requiring fewer reagents. The resulting methods were rapidly deployed in the university laboratories, and subsequently, pharmaceutical companies and suppliers made available their laboratory space, people, reagents, and instrumentation [3]. The Belgian government had called on all universities, research centers, and industry to make instruments available for a period of 6 months.

Industry partnerships on information technology were set up with providers UgenTec and CliniSys|MIPS. UgenTec provided a software solution for automated data analysis and interpretation, distributed high-volume result calling, qPCR result quality control (QC), dashboarding and integration for proficiency testing. CliniSys|MIPS provided an integrated Lab Information Management System (LIMS) to facilitate sample accessioning and tracking the sample routing across partners and integrate sample reporting into the national health databases.

### Setting Up a National Testing Initiative: A Distributed Approach

Large-scale testing initiatives were being set up internationally [4,5,6,7,8], and the need for collaboration between clinical laboratories, public health agencies, and industry to control the outbreak quickly became apparent [9]. Strategic planning for the Belgian national initiative began with a logistics assessment of available resources, including testing facilities, availability of assay kits or component reagents, instrumentation, and consumables. To support logistics assessment, Thermo Fisher Scientific (TMO) joined the Belgian testing consortium as an assay and instrumentation manufacturer and was able to provide an overview of the available and applicable instrument installed base within the country that could be mobilized for the initiative.

Because the national lockdown was already in place at that point in time, several testing projects across industries and applications were already on hold (freeing up instruments, personnel/volunteers, and time), which limited the impact on ongoing private and academic testing initiatives, facilitated instrument hand-over, and allowed participating groups to contribute to emergency public health needs, in times of infrastructure and supply chain stress and scarcity.

With the aid of the Belgian army, the owners of available instrumentation were contacted to repurpose their systems for the national testing program. The instruments were then collected and redistributed to four of the five testing sites, in accordance with the instrument qualification standards required for diagnostic laboratories.

These testing sites, Biogazelle NV, GlaxoSmithKline Biologicals SA, Janssen Pharmaceutica NV, UCB Biopharma SRL, and University of Liège, joined forces under the clinical and quality assurance supervision of the Belgian NRC for Respiratory Pathogens at the University Hospitals of Leuven. The five testing hubs primarily comprised pharmaceutical research and testing service laboratory sites which were outfitted with instrumentation collected and compiled from any available existing systems already installed within the country and had worked together on the automation of processes.

A key consideration was for the initiative to be set up rapidly, so upon checking the available resources with potential partners, it was the academic and pharma partners that had instrumentation available that would be able to be repurposed on very short notice, utilizing capacity that was or had become idle because of research initiatives slowing down or halting. The second consideration was the willingness of partners to free up human resources on a short term. Thirdly, the required expertise was present with the partners that volunteered to participate: experience in molecular diagnostic testing, scale-up, robotization, and automation.

Early on, the consortium focused on instrument and assay selection and validation. Once instruments for RNA extraction and qPCR analysis were moved to the participating testing sites, they underwent a maintenance checkup by the suppliers. Once completion, the laboratories organized for high-throughput testing put staff training initiatives in place and started the required assay and personnel validation activities.

Two commercial lab services companies, BARC and PPD, supported the central lab activities with sample logistics. By April 2020, this consortium was essentially devoted to a large campaign of testing in homes for older people and retirement homes, launched by the federal and federated entities [3].

## 2. Rapid Multi-Site Setup Using Available, Heterogeneous Resources

With platform and assay selection limited by available resources, the partnering laboratories made use of a variety of assays, protocols, controls, liquid handling, and thermocycler instrumentation. This significantly shortened the time from validation to routine testing and helped bring capacity online within weeks.

Moreover, with stringent requirements on timeline to deployment, the task force opted to allow the participating partners to retain control over their respective personnel and assay validation processes, standard operating procedures, and lab protocols of choice. This allowed accelerating the implementation of a sizeable emergency testing capacity.

To ensure continuity and mitigate supply chain risks, readily available but heterogeneous assays were selected. For example, one testing site deliberately opted for alternative instrumentation and consumables for every step of the workflow, while still linking into the centralized data analysis processes. Table 1 summarizes instrumentation and assay details per participating testing site.

Once shortages in commercially available reagents became apparent, the academic partners mitigated the risk of several months of delays by designing, validating, and manufacturing extraction kits based on available materials and proprietary expertise.

Consecutively, when supply chain issues eased, labs continued to rely on multiple technologies in order to maintain robustness against future supply chain gaps.

## 3. Assay and Instrument Selection and Validation

**Figure 1:**
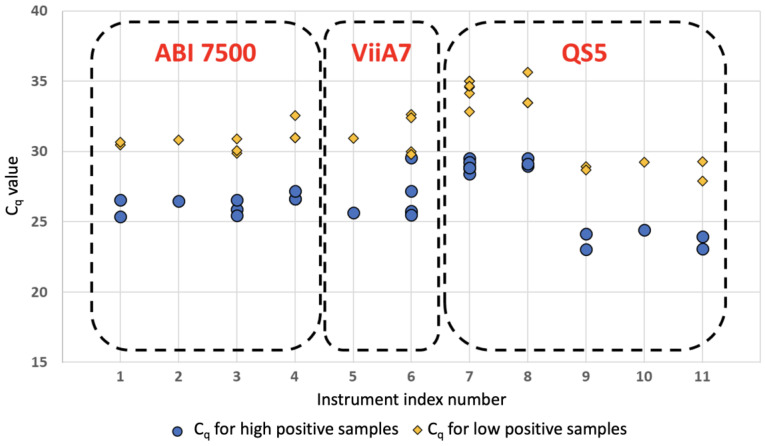
*Analysis of the N gene across multiple sites and instruments*. The data set used was a limited sample of validation data for a period of 10 days in July 2020. While not representative of the post-validation workflow, the analysis illustrates variation across instrumentation and site, which needs to be taken into account in data analysis and reporting.

Underscoring the importance of instrument and assay validation, a significant number of qualification panel samples were run prior to going live and were analyzed across instruments and laboratory participants. Figure 1 shows an analysis of the N gene for those samples across multiple sites and instruments. In yellow, low positive C_q_ values are shown. Aggregate visualizations provide an overview across sites and instruments for the N gene marker.

The data set used was a limited sample of validation data for a period of 10 days in July 2020. The analysis illustrated variation across instrumentation and site, which needs to be taken into account during data analysis and reporting. The analysis illustrated the complexity and heterogeneity of the data and the importance of consolidation. Without proper consolidation tools, maintaining the necessary quality would prove to be a herculean task.

## 4. Logistics and Sample Flow

**Figure 2:**
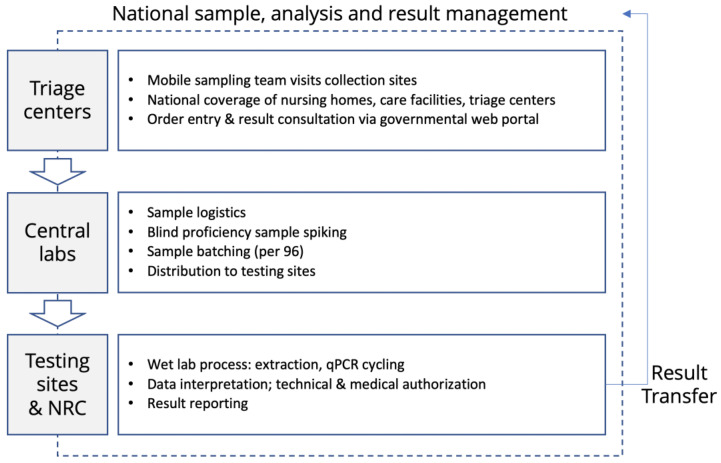
*Overview of logistics and sample flow* shows the flow from sampling location to the testing site. The sample logistics and the related sample accession and result data flow are organized based on a single and unique sample number, which makes the data flow compliant to Global Data Privacy Requirements and facilitates sample chain of custody, traceability across the multiple sites, and centralized result reporting through a governmental health portal.

Figure 2 summarizes the flow of samples from collection sites over triage sites to the central labs, where batching and blind QC panel addition is performed. From there on, samples are distributed to the participating sites, and results are returned to the requesting physicians and facilities.

Of note, data were anonymized, so that no patient and subject information was made available to any of the testing labs.

## 5. Quality Assurance of COVID-19 Testing—Continuous QC Monitoring

A continuous proficiency testing program, managed and overseen by the Belgian NRC at the University Hospitals of Leuven, was implemented to monitor and maintain the quality of the tests once they started testing across the five testing sites.

Before a test site could start testing clinical samples, the laboratories needed to successfully classify a set of blinded samples. This qualification panel of 91 samples, prepared and tested beforehand by the NRC, was created to provide information about the sensitivity, intra-run reproducibility, robustness, and variation between the C_q_ values of the internal controls. In case changes were made in the testing procedure on a particular site, a re-evaluation was performed with a panel prepared by the NRC.

MIQE guidelines [11] recognize the use of C_q_ as the optimal way to report qPCR results across different platforms. Of note, software-configured plugins representing various assay–instrument combinations were used to parameterize and standardize the various result values to a single representative test outcome.

### 5.1. Setup of the QC Panel

Per 940 patient samples, one QC panel consisting of one strongly SARS-CoV-2-positive, a single weakly SARS-CoV-2-positive, and one (three, from mid-August 2020 onwards) SARS-CoV-2-negative QC sample were added. QC panels were produced by NRC UZ Leuven—building on their existing ISO 15189 accreditation. These QC samples were randomly and blindly placed among the patient samples daily by the distributor centers before sending the batches off to the laboratories for analysis.

The NRC evaluated and monitored QC sample results in real time and on a daily basis, using the LIMS system set up for this consortium and UgenTec’s FastFinder Insights tracking dashboards and export capabilities to evaluate the performance of individual laboratories. The testing sites were expected to pass daily proficiency testing by meeting performance specifications for the proficiency testing. If a testing site failed the proficiency monitoring program, it was required to evaluate with the NRC if there was a need to halt sample processing and implement the necessary corrective and preventative actions before restarting its testing activities.

### 5.2. Logistics for Sample Preparation, Distribution, and Nonconformance Monitoring

Using a strongly SARS-CoV-2-positive sample, NRC UZ Leuven prepared strongly and weakly positive QC samples. Negative QC samples only contained viral transport medium. The QC samples were prepared in sampling tubes that were visually undistinguishable from patient samples, and to each sample a swab was added. The QC samples were registered into the central LIMS system in an identical manner to that of regular patient samples. Next, QC samples were transported to the two centralized distribution hubs, one for the north of the country (Flanders) and one for the south (Wallonia), responsible for the logistics, where they were kept and stored at −80 °C. These central hubs were tasked with collecting patient samples from the various collection sites (such as triage centers, nursing homes, and prisons) and to distribute the workload among the participating laboratories. The aggregated patient samples were boxed for transport, and the Central Laboratory included the QC panel. The sample boxes, including the randomly placed QC samples, would then be distributed to the testing facilities for normal processing.

The following day, the NRC at Leuven University collected the QC sample results from the LIMS system of this consortium and UgenTec’s FastFinder Insights tracking dashboards and assessed the correctness of calls. The results of the previous day’s blinded QC samples, which were reported as a qualitative test result (Positive, Negative, or inconclusive), were relayed back to the participating laboratories before 10 AM. In case of discrepancies, the laboratories were required to open a procedure for nonconformity in their respective quality management system to identify the root cause, and a mitigation meeting was set up between the appropriate laboratory, Sciensano, and NRC to initiate analysis, determine whether the laboratory could process that day’s batches of tests, and define corrective and preventive actions when required.

## 6. Information Flow through the Initiative’s Informatics Platform

The ability to directly connect laboratory result data to national academic reference centers and national public health surveillance systems is crucial in the control of COVID-19 [12]. Positive rates were tracked on a daily basis at the NRC. Moreover, the NRC was closely involved in the coordination of the national testing initiative and had access to all data to follow up on sample volumes and other key metrics such as result statistics and continuous QC monitoring metrics. A real-time dashboard was available for each partner to monitor their sample flow, data analysis, and result reporting; insights into sample flows, key QC metrics, and test positivity rates were available to the consortium.

**Figure 3:**
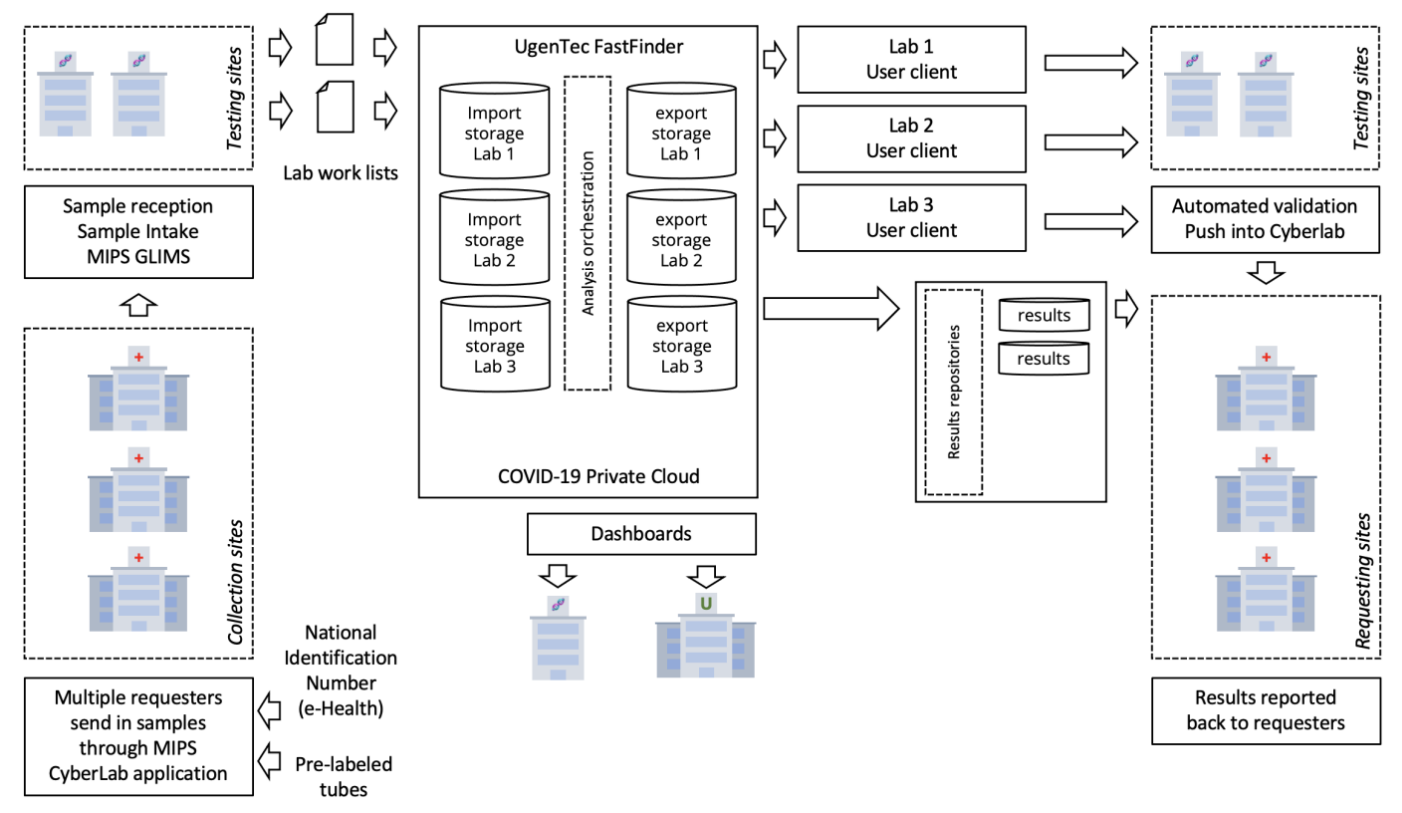
*Data flow through the IT systems.* At sample collection sites (**bottom left**), national identification and pre-labeled tubes are made known to the system. HL7 connectivity is used to create sample work lists for the testing sites (**top left**). Work lists and raw test results from cycler instrumentation are ingested by the interpretation and result generation software. Environments and clients are available per tenant, and dashboards for QC are available to the NRC. Called results go back to the requesting sites (**bottom right**), with restricted views per requester. Results immediately flow back to the requesting physicians and patients.

Figure 3 shows the flow of samples from sampling locations to test sites. The informatic for sample registration and tracking, data analysis interpretation, result generation and reporting, and QC tracking was set up through industry partnerships.

## 7. Industry Partnerships for Information Management at Scale

A high demand for testing increases the administrative burden on lab staff and puts specific requirements on informatics systems managing test ordering, registration, sample flow, and result reporting [13]. The multi-site nature of the initiative and the lack of cross-site instrument and assay standardization added additional requirements to the program.

- To allow for results to be consistent across sites and reporting to be standardized, a data pipeline with the ability to account for cross-site variability needed to be put in place to capture the heterogeneous assay result files from the thermocyclers and process the raw qPCR data to standardized, reproducible results across partners, while, at the same time, supporting continuous result QC.
- Moreover, to facilitate sample accessioning and full traceability across (a) participating laboratories, (b) sample collection and distribution centers, and (c) the clinical and quality assurance supervision of the Belgian NRC for Respiratory Pathogens at Leuven University, a central accessioning and sample tracking Lab Information Management System (LIMS) needed to be put in place, serving as a central data hub across the consortium.

To support the initiative, the government task force selected commercial IT and bioinformatics service providers to setup the information technology infrastructure. CliniSys|MIPS was selected for the implementation of a national LIMS system across all testing sites and implemented their GLIMS product for lab workflow management within the respective test sites. Additionally, their CyberLab product was implemented outside of the testing laboratories to allow all sample collection sites to create test orders digitally and provide an order entry and management system. As soon as the test results were validated in the LIMS system, they were made available through standard HL7 connectivity to the CyberLab web-based result portal.

UgenTec was selected for the harmonized data analysis pipeline across all instruments, assays, and sites, based on their FastFinder platform. FastFinder Analysis serves as the laboratory facing component for automated analysis and result generation. FastFinder Insights provides dashboards for QC monitoring metrics, positivity rates, and trend tracking.

Both software solutions where securely hosted in the Microsoft Azure cloud and had a direct and real-time interface for result transfer. The platform allowed for result data to immediately be made available to the requesting physician, as well as the patient. This happened through a real-time integration with the Belgian national e-Health platform, a portal for exchange of health information between citizens and providers.

### 7.1. A Standardized Analysis and Reporting System

Comparable to the need for automated extraction robotics or automated liquid handling, automated analysis software was a key requirement to fulfill the national testing throughput, turnaround times, result consistency, and quality objectives. With the various individual thermocycler software tools that come with the instrumentation being tailored to their respective vendor instruments, there was an unmet need for a platform agnostic software solution to support each of the data analysis pipelines across the consortium.

Early conversations with participating laboratories identified that standard instrument software associated with thermal cyclers would fall short of high-throughput analysis requirements due to the need for manual reviews by molecular experts. Manual analysis, review, and report generation on the qPCR data were not feasible with the large sample volumes collected in the Belgium COVID-19 testing initiative due to the high sample volume (daily peaks above 10,000 samples were not uncommon).

Moreover, with a heterogeneous fleet of instruments recruited into the consortium, the lack of singular interpretation operating procedures would negatively impact result standardization across sites. Some laboratories complement instrument software with research software pipelines that are built internally, but these are not fit for large-scale diagnostic use because they typically lack locked algorithms and data analysis parameters that guarantee reproducibility and standardized data analysis.

Additionally, curve calling, thresholding, and interpretation rules differ across and are specific to the assays used. Moreover, result values are impacted by instrumentation, chemistry, marker selection, type of positive and negative controls, use of replicates, etc. These parameters were specific per assay, instrument, and site. Hence, result calling needed to be tailored per assay and instrument type. To this end, the software platform for analysis and interpretation represents qPCR curve calling and assay decision logic in small, immutable packages with a versioned configuration and logic—called Assay Plugins.

**Figure 4:**
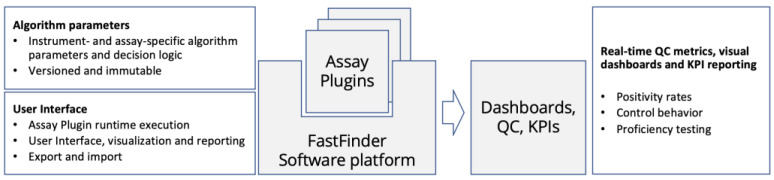
*A layered software architecture.* Assay logic and curve-calling algorithm (Assay Plugins) are separated from the execution and visualization framework, and laboratory dashboarding is decoupled from the platform database.

Figure 4 visualizes the software architecture of the data analysis solution. Assay Plugins are versioned representations of PCR curve calling and assay interpretation logic, specific for individual assays and instruments used. Plugins are versioned separately from the analysis software. This allows consistent data analysis across consecutive versions of the analysis software, which needs to evolve rapidly to meet the changing needs in a deployment at scale. For example, the user interface, systems integrations, dashboard visualizations, and external interactions evolve and are updated separately from the assays.

Broader advantages of the architectural separation of assay logic and user interface become evident with the need for rapid software changes and additions during initial product ramp-up, optimizing peripheral functions such as QC metrics, LIMS integration, reporting capabilities, visualization tools, accessioning information import, connectivity to instrumentation, scripted file consumption and generation, etc. As an example, the approach has proven valuable to support updates in reporting capabilities and the addition of safeguards such as implementation of a cross-contamination checking functionality without requiring Assay Plugin changes or indeed without impacting results and clinical validation.

### 7.2. Monitoring Tools and Dashboards

Real-time laboratory operational intelligence is critical for maintaining an overview of systemwide functionality and performance, especially in the exceptional circumstances of a pandemic. The software component for QC dashboarding was deployed (Figure 4) as a separate component to avoid impacting the performance of the routine platform critical to high-throughput operations. Dashboards were implemented to provide live visibility to critical operations data such as test turnaround times across the instrument fleet, instrument reliability performance, assay and plate validity, test outcome rates (positivity rates, void result rates), and QC metrics such as statistics on neighborhood effects on well plates.

To maintain an overview of daily sample volumes, positivity rates, instrument and control behavior, and QC metrics, cross-site report dashboards were set up on the FastFinder Insights platform. FastFinder insights aggregates assay, result, and workflow data across all participating laboratories and provides a database and reporting infrastructure allowing for configurable views, report exports, and follow-up of key metrics.

**Figure 5:**
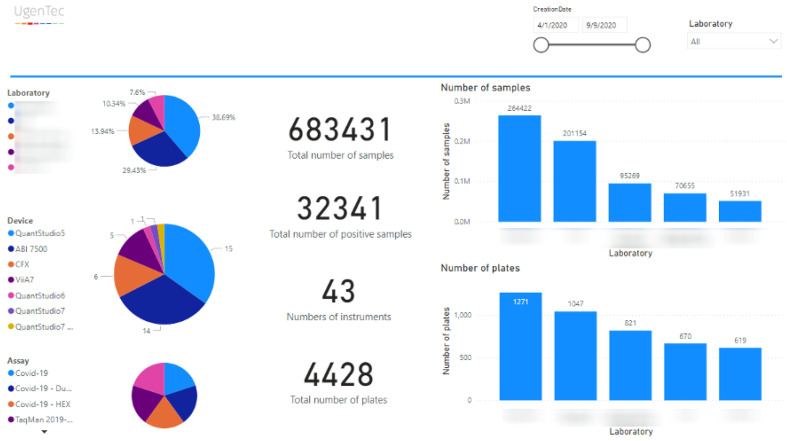
*FastFinder Insights*. A dashboard overview with key metrics across participating laboratories, devices, and assays.

Figure 5 shows a dashboard overview for a specific date range, highlighting key metrics across participating laboratories, device types in use, and assays deployed. Sample counts, positive counts, and plate, run, and sample statistics are visualized in a single dashboard. The overview contains the key metrics configured as overview dashboard across participating laboratories for the first 6 months of production testing.

**Figure 6:**
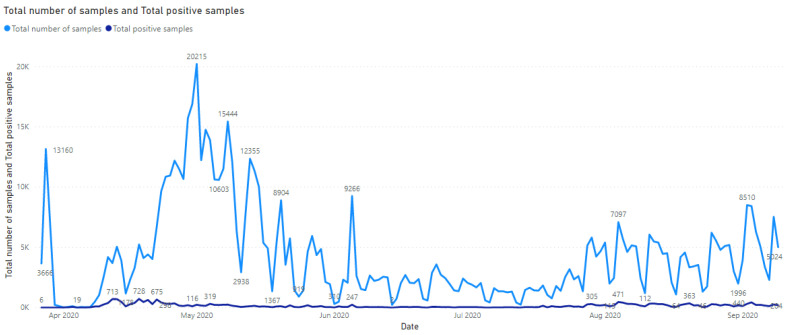
*Trend analysis.* Time series of the total samples and positivity rates. Interactive visualization available in the FastFinder Insights software user interface.

Detailed reports were set up to track individual key performance indicators. As an example, Figure 6 shows a time series of the total number of samples processed across the five testing laboratories versus the positivity rate (samples identified as SARS-CoV-2-positive by qPCR).

This architecture provides a framework for validated assay interpretation logic, while still maintaining flexibility for rapid release cycle updates and benefiting from agile software development. Additionally, a hosted software infrastructure also reduces strain on laboratory personnel who, themselves, are affected by the lockdown measures implemented for the broader public: with samples being processed in shifts across multiple runs, staff need not wait until completion of the last run of the day but can review and sign off results from home, shortening their time in the laboratory.

## 8. Turnaround Times

The consortium approach, with distributed lab sites, each processing a part of the test volume, allowed results to turn around within a day. A consortium setup was the only viable approach early in the pandemic and with a centralized structure, TaT cannot be reduced beyond the point of limitation determined by sample transport and logistics.

### 8.1. Guaranteeing Results within a Day

The turnaround of 1 day mandated by the governmental working group is linked to how collection logistics were set up, as well as how labs process samples. The participating labs were running their workflow with daily cutoffs. Typically, a sample would have to arrive before 8 AM for it to be released the same day. When retesting was required, the result release would not happen before the retest was performed. Equally, if there was doubt about the result, sample analysis would be repeated. In addition to retesting, samples that would arrive during the day would be moved into the next day’s batch.

Within the lab workflow, a sample TaT of 12 h was attained for the samples that fit the schedule—i.e., met the cutoff deadline and did not require re-testing.

The number of repeats showed dependence on the positive rate of testing. The presence of large numbers of highly positives in the labs’ batches was impactful, as it increased the probability of intraplate contamination.

As to the logistics prior to a sample arriving at the labs, the capability to scale was built into the approach by engaging experienced logistics partners to bring the samples from the de-centralized collection locations to the central locations.

### 8.2. Maintaining TaT with Growing Volumes

The consortium’s testing initiative did not comprise the entire country’s capacity—rather, separate initiatives (e.g., hospital and private labs) were active in parallel. Testing capacity and TaT were maintained through initially limiting consortium testing to symptomatic patients in high-risk centers such as elder care facilities, nursing homes, and correction facilities. Collection sites and inclusion criteria broadened as the consortium set out to ramp up capacity over time—doubling from 2000 to 4000 by summer and growing from there onwards—which was made possible through implementation of automation and robotics.

With sample volumes increasing and robotics brought in place, new strategies were needed to avoid that turnaround times would be negatively impacted by issues such as cross-contamination caused by increased positives’ rates (highly positive wells possibly impacting nearby plate positions).

To mitigate those newly arising issues, software updates were put in place to detect cross-contamination automatically. Here, the benefit of decoupling analysis automation from peripheral software functions such as dashboarding, monitoring, and reporting allowed new tools to be deployed to automatically identify samples that potentially were affected due to proximity to highly positive wells and needed to be flagged for retest, maintaining TaT for unaffected samples.

Now, well into the pandemic, decentralized testing initiatives, reinforcement of regional networks of clinical laboratories, installation of mobile laboratories [14] and point-of-care testing [15,16], and the adoption of different laboratory technologies [15,17,18,19] will allow to further reduce TaT, and recent initiatives will soon lead to turnaround times below the hour.

## 9. Lessons Learned

The initial supply shortages created significant challenges to get testing infrastructure up and running in a timely fashion. The consortium worked with instrumentation, protocols, kits, and reagents that were available at the time to de-risk dependency on overseas supply chains and developed their own reagents and methods where required. The lack of standardization and increased complexity were mitigated by a twofold approach:

- Continuous, comprehensive QC program—on a daily basis, blind control samples were introduced in every batch distributed to all participating labs. The blind samples contained negatives, weakly positives, and strongly positives. Every morning, labs were permitted to continue testing only if no QC issues had come up with the blind control samples.
- AI-based result-calling software tailored to heterogeneous assays –harmonization of assay results being a key requirement, a validated calling software approach was tailored to each lab’s combination of assay, instrumentation, and protocols to ensure uniform positive/negative result calling. The calling algorithms and interpretation decision logic were represented in locked versioned plugins, allowing the user interface and high-throughput tooling to evolve without impacting the outcome or result calling.

The consortium ramped up quickly and mitigated supply chain issues by allowing for different standards but compensated for the effects with (a) a robust QC program that checked results on a daily basis and (b) a software strategy to ensure harmonization of result calling.

A playbook that mitigates dependency on commercial suppliers through self-sufficiency in provision and procurement of materials and instrumentation and by harmonizing through QC and analysis strategies ensures a better preparedness for consecutive waves and future pandemics.

## 10. Conclusions

The rapid scale-up of a national qPCR-based SARS-CoV-2 testing initiative across multiple testing sites required the recruitment of resources as they were available, resulting in a heterogeneous collection of sample flows, instruments, SOPs, software tools, LIMS systems, and analytical tests. Integral proficiency testing and integrative single software data analytics and result reporting infrastructure were essential to ensure consistent, reliable results at a high daily sample volume, peaking above 10,000 results per day.

To standardize performance tracking and monitor and maintain quality across the five testing sites, a blinded proficiency testing program was implemented in the workflow using the LIMS system of this consortium and UgenTec’s FastFinder Insights tracking dashboards. Proficiency testing on an ongoing basis allows QC issues to be identified quickly, and a hub-and-spoke distribution model supports optimal resource allocation across multiple high-capacity test sites.

Integrated informatics tools for sample tracking, reporting systems integration, data analysis standardization, and interpretation support automation based on a layered architecture allow for rapid adaptations in an environment where technical and medical validation are drastically shortened.

A cloud-based approach with instrument integration into automated analysis and sign-off allowed laboratories to realize same-day reporting on almost all of the tests through reduced time to result, without additional workload burden on critical workers and allowing a reduction of their presence in the laboratory through remote data approval.

To summarize, in normal circumstances outside of a global pandemic, a stable laboratory environment, established logistics and supply chains, known and established instrumentation and protocols, and extensive validation periods allow for robust implementation of new assays. However, under emergency circumstances, the need for a rapid increase in sample volume, the constraint of working with available heterogeneous instrumentation, monitoring the sample flow and assuring the quality of the COVID-19 tests, as well as additional unknowns in workflow and integration at the start of the initiative, make a tailor-made computer support with good software necessary.

The consortium was able to rapidly develop their laboratories into high-throughput testing sites despite the diversity of approaches and time pressure, with proven effectiveness and efficiency in terms of assay reproducibility, assay confidence, quality controls, and turn-around times. Our approach to quickly scale up nation-wide COVID-19 testing during an ongoing pandemic, filling an emergency need, was made possible by using state-of-the-art technologies and tools and was successful only because of the commitment, dedication, and passion of hundreds of scientists and volunteers from the various institutions involved.

## Figures and Tables

**Table 1:** Overview of participating testing sites and instrumentation and assays in use.

**Partner**	**RNA Extraction**	**qPCR Instrumentation**	**Assay**	**Target Genes**	**Controls**
Biogazelle	96-well filter plates (Norgen Biotek #24380, Zymo Research #R1041)	Bio-Rad CFX384	LDT based on the Charité [10] E gene assay; proprietary SIC; PrimeScript III (Takara) & iTaq (Bio-Rad) one-step RT-PCR mix	duplex 1 well, E & SIC	PC (Twist Biosciences SARS-CoV-2 RNA + diluted transport medium) and 2 full workflow NC (transport buffer)
UCB Pharma	KingFisher; U Liège extraction buffer	Thermo Fisher QS3, ABI 7500, ViiA7	Thermo Fisher TaqPath COVID-19 CE-IVD RT-PCR Kit	multiplex, MS2, ORF1ab, N, S	Thermo Fisher
Janssen Pharmaceutica	96-well filter plates (Norgen Biotek #24380)	ThermoFisher QS3, QS5, QS7, QS6, QS7 Pro	Thermo Fisher TaqPath COVID-19 CE-IVD RT-PCR Kit	multiplex, MS2, ORF1ab, N, S	Thermo Fisher
GSK	KingFisher; U Liège extraction buffer	Thermo Fisher ABI 7500, ViiA7	Thermo Fisher TaqPath COVID-19 CE-IVD RT-PCR Kit	multiplex, MS2, ORF1ab, N, S	Thermo Fisher
U Liège	Magnetic beads RNA isolation	Thermo Fisher QS5, Bio-Rad CFX384	Thermo Fisher TaqPath COVID-19 CE-IVD RT-PCR Kit	multiplex, MS2, ORF1ab, N, S	Thermo Fisher

## Abbreviations and Acronyms

Abbreviations and Acronyms used in the manuscript.

**Abbreviation**	**Meaning**
QC	Quality Control
C_q_	Quantification cycle
IT	Information Technology
COVID-19	Coronavirus disease
SARS-CoV-2	Severe Acute Respiratory Syndrome Coronavirus-2
NRC	National Reference Center for Respiratory Pathogens
FAMHP	Federal Agency for Medicines and Health Products
HL7	Health Level Seven global health data interoperability
ISO	International Organization for Standardization
DNA	Desoxyribonucleic Acid
RNA	Ribonucleic Acid
LIMS	Lab Information Management System
LIM	Lab Information Management
CE-IVD	Conformité Européenne-in vitro Diagnostic
RT-PCR	Reverse-Transcription PCR
PCR	Polymerase Chain Reaction
qPCR	Quantitative PCR
SOP	Standard Operating Procedure
TAT	Turnaround Time
WHO	World Health Organization
SIC	Spike In Control
PC	Positive control
NC	Negative control
